# Age-related differences and discrepancies between objective and self-assessed executive functions in youth academy footballers

**DOI:** 10.3389/fspor.2026.1711349

**Published:** 2026-07-09

**Authors:** Levente József Szántai, Tamás Berki, László Tóth

**Affiliations:** 1 Doctoral School of Sport Sciences, Hungarian University of Sport Sciences, Budapest, Hungary; 2 Department of Physical Education Theory and Teaching Methodology, Hungarian University of Sport Sciences, Budapest, Hungary; 3 Department of Psychology and Sports Psychology, Hungarian University of Sports Science, Budapest, Hungary; 4 Teacher Training Institute, Hungarian University of Sports Science, Budapest, Hungary; 5 Institute of Health Promotion and Sport Sciences, Faculty of Education and Psychology, Eötvös Loránd University, Budapest, Hungary

**Keywords:** age groups, executive functions, football, self-assessment, sport performance

## Abstract

**Introduction:**

Football is characterized by rapidly changing game situations in which executive functions (EFs) are crucial for successful mental and physical performance. Previous research has highlighted the importance of cognitive abilities in decision-making and performance; however, the examination of discrepancies between objectively measured executive functions and subjective self-assessments among young players of different ages is still a relatively unexplored area.

**Methods:**

Participants were 102 players from a Hungarian football academy, divided into five age group (U14, U15, U16, U17, U19). EFs were assessed using computer-based tools from the Vienna Test System (VTS), including reaction time (RT), stress tolerance (DT), visual perception (LVT), and focused attention (SIGNAL). Self-assessment was measured using study-specific questions designed to assess players perceived executive-function-related abilities in sport contexts, while the final ranking of the teams in the league and their average goals scored per league match were taken into account as descriptive team metrics.

**Results:**

Age-related differences in EF performance were observed. The U19 group achieved the best results on the SIGNAL and LVT tests, indicated that the oldest players had the highest concentration ability and were best able to filter out relevant visual stimuli. The U17 group performed best on the RT, showing the fastest motor response. Furthermore, the U16 performed best on the DT, demonstrating the highest level of stress tolerance. Regarding subjective self-assessment, the only significant between-group difference was observed in the LVT, where the U19 rated themselves highest in terms of filtering out relevant visual stimuli. Finally, in all groups, players’ subjective self-assessments were higher than their EF scores measured by objective tests.

**Discussion:**

Findings partially confirmed cognitive differences between age groups in objectively measured EFs. It is important to note that, in this sample, subjective self-assessments of cognitive abilities differed from the results of objective measures of executive functions. These findings can serve as a guide for future research examining the discrepancy between cognitive abilities and self-assessment. Professional feedback about the level of cognitive abilities supports the development of a realistic self-image, integrated into complex development programs designed for players. The results are specific to one academy; in terms of generalizability, it would be worthwhile to include other academies in future studies.

## 1 Introduction

Football is characterized by rapid changes in game situations, where the players’ executive functions (EFs) are key to successful performance (1–4). It can be considered a complex cognitive construct (5) that is fundamental to maintaining attention, inhibiting inappropriate stressful environmental stimuli (6), and flexible adaptation to changing situations (7). EFs include three core components: working memory, which enables the storage and manipulation of visual and spatial information; inhibition, which involves self-control, selective attention, and the suppression of inappropriate behaviours; and cognitive flexibility, which refers to the ability to switch quickly between tasks and strategies (6, 8, 9). These components form the foundation for higher-order EF processes, including reasoning, planning, and problem solving (10). The relationship between EFs and physical activity provides important opportunities for examining cognitive functioning in sport contexts.

Physical activity has been shown to have positive effects on EFs, attention, and academic performance in children aged 6–12 years (1). Intense physical activity also improves cognitive performance by enhancing attentional focus (2, 3). In the long term, the positive effect of physical activity on attention increases children's engagement with school-related tasks, thereby contributing to better academic performance (4). Vigilance refers to the ability to maintain attention over the long term and respond appropriately to relevant stimuli. There is a positive association between regular participation in sports and vigilance in adolescence (11). Regular participation in externally paced sports, such as football, significantly improves vigilance, reaction time and inhibitory control, compared to self-paced sports, such as triathlon, and in adolescents with a sedentary lifestyle (12, 13). Physical activity involving higher cognitive effort has a greater impact on EFs than aerobic physical activity (5). Programs aimed at physical activity have a positive effect on working memory and cognitive flexibility, in contrast to inhibitory functions (6–8). Furthermore, physical fitness also has a positive effect on short-term working memory, and this effect is even more marked in older elementary school children (9). Therefore, externally paced sport environments that combine high physical, perceptual, and cognitive demands provide an appropriate context for supporting cognitive development during adolescence (14). In this regard, football represents a particularly relevant setting for examining the development of executive functions in youth athletes (14).

Football can be considered an intense physical activity in which EFs appear specifically in dynamically changing game situations through decision-making, adaptation and the application of appropriate strategies. During play, a players processes and stores information from various game situations (1), filters out irrelevant stimuli, recognizes and recalls the strategy necessary for an adequate solution, and then applies it to decision-making in a fraction of a second (4, 15). For example, when a player receives the ball from a teammate, he must quickly scan the pitch to identify the best passing option. In this situation, the player must ignore teammates who are tightly marked by opponents and select a teammate who is free and ready to receive the pass. Based on the reviewed literature, the present study aimed to examine EFs in football, as these cognitive abilities are increasingly recognized as relevant indicators of sports performance (1, 15, 16).

EFs are positively associated with sports performance. In football, young athletes have been shown to demonstrate higher inhibitory function and cognitive flexibility than individuals who participate at a lower level or do not compete (17, 18). Talented young players have better motor inhibition and attention-shifting abilities, which are essential for adapting to dynamic game situations (19, 20). Furthermore, visual orientation has a significant impact on sports performance, which is based on effective working memory function (21). Higher EFs scores have also been linked to performance-related indicators, such as goals scored and team league ranking. Overall, previous studies confirm the significant associations between EFs and sports performance. In addition, age appears an important factor that plays a key role in the development of EFs among athletes (17, 18, 22).

EFs generally develop as children get older, and experience in football further supports this developmental process (23, 24). Among elite youth players, both age and football experience play an important role in long-term sustained attention and in the ability to switch between tasks that arise in game situations (23, 25). Differences in EFs across age groups are therefore partly explained by cognitive maturation, as older players generally show stronger inhibitory control and cognitive flexibility (22, 26). At the same time, although EFs are related to age, more marked developmental changes are observed in younger players, suggesting that the development of EF components may plateau around the end of adolescence (26). In addition to age an important factor influencing EFs is the athlete's realistic assessment of their own abilities (23, 24, 26).

Self-assessment in sport refers to the process during which athletes evaluate their performance based on specific criteria, which can improve their self-awareness, self-regulation and critical thinking (27–29). Self-assessment regarding skills affects athletes’ decision-making and planning, which in turn can impact the effective functioning of EFs (30, 31). During stressful situations, emotional control and focused attention are essential for effective decision-making, influencing athletes’ self-regulatory behaviour. These cognitive strategies are key to effective self-assessment and overall success in sport (32).

Young players who realistically assess their own performance progress more effectively toward their adult sports careers, as they are better able to identify their strengths and areas for improvement and focus their efforts accordingly (33). However, overly positive self-assessment can lead to an overestimation of players’ abilities, which negatively affects their sports performance and could increase anxiety (33). In addition, coaches’ subjective perceptions can also distort the ranking of players’ performance levels. It is therefore important to also consider the effectiveness of physical objective measurements such as jumping ability, sprint speed, and change of direction when ranking players on their performance levels (10, 16). Research suggests that objectively measuring executive functions, such as inhibitory control and cognitive flexibility, is a more accurate predictor of young players’ performance than self-assessment (18, 26).

Building on previous research, this study aimed to examine differences in EFs across age groups and discrepancies between subjective self-assessment and objective EF measurements in a Hungarian sample of young football players. Although EFs and self-assessment are considered important in youth football development, differences between perceived and objectively measured EF-related abilities have not yet been systematically examined across age groups. Accordingly, the study had two primary objectives: First, we aimed to examine discrepancies between self-assessed and objectively measured EFs. Second, we aimed to investigate how both self-assessed and objectively measured EFs differ across age groups.

## 2 Material and methods

### 2.1 Participants and procedure

The U14, U15, U16, U17 and U19 age groups of a Hungarian football academy participated in the study (*N* = 102; M = 16.2 years; SD = 2.07). According to the rules of the Hungarian Football Association, there is no U18 age group, so players who belong to this age category can play in the U19 team for two years (34). The sample included age groups in which members had at least five years of football experience, trained five times a week, and played matches in the Hungarian youth first division on weekends. Additional criteria included the same pitch size, the same competition level and training methods based on the same professional principles as an additional factor influencing EFs. Four players from the U17 age group were not included in the sample because they had stopped playing football or were unable to participate in training for more than three months due to serious injuries. The teams’ characteristics—including average goals scored per match and final season rankings—are shown in (Table 1).

**Table 1:** Team indicators of the study variables.

Groups	N	Average number of goals scored	Place at the end of the season
U14	19	3.42	1
U15	23	2.23	3
U16	23	1.92	4
U17	13	1.35	4
U19	24	2.52	3

The EFs tests and self-assessment EFs were performed between June and July 2024, before the afternoon training sessions during the summer football pre-season. Participants first answered self-assessment questions about EFs and then completed the Vienna Test System (VTS) tests (3). The pre-season assessment window was selected because it allowed standardized testing across all age groups under comparable organizational conditions; during the competitive season, data collection was substantially constrained by school attendance, match schedules, and regular training demands. The all the tests took 45 min–50 min to complete and were performed in an air-conditioned room with the consent of the players. We requested prior informed consent from parents for participants under the age of 18 and permission from the academy's professional director to conduct the research. The research was conducted with the permission of the Ethics Committee of the Hungarian University of Sports Science (MTSE-OKE-KEB/04/2023).

Computerized tests using VTS were conducted to measure EFs; this is a widely used computerized testing system for measurement of cognitive abilities in various sports (35, 36). It is considered a reliable and valid psychometric tool, with reliability indicators for the tests used ranging from 0.84 to 0.95 as previous studies stated (37). In this study, general EFs were measured in the context of football. Reaction Time Test was applied (RT) for motor reaction time measurement, Determination Test (DT) for stress tolerance, Signal Detection Test (SIGNAL) for long-term sustained attention, and Visual Perception Test (LVT) for visual perception. The raw data from the tests was converted by VTS into values ranging from 0 to 100, making them comparable in terms of percentage to the results of the self-assessment.

### 2.2 Measures

#### 2.2.1 Reaction time test (RT)

During the RT, 28 yellow light stimuli appear on the computer screen at various random intervals (2.5 s–6.0 s). Participants press a reaction key as quickly as possible in response to the programmed visual stimuli. If a light of a different color appears on the screen, the participant's task is to keep their finger on the readiness key, thus inhibiting any response to the incorrect light.

#### 2.2.2 Determination test (DT)

DT measures responsiveness to complex stimuli and is therefore related to the ability to switch between tasks and to stress tolerance. Participants must respond to visual stimuli (white, yellow, red, green and blue colors) and auditory stimuli (high and low tones) by pressing the button belonging to the stimulus. In addition, they can press one of the two reaction pedals with their right or left foot in response to white lights appearing on a black background on the screen.

#### 2.2.3 Visual perception test (LVT)

The LVT measures visual orientation performance in complex environments, which is related to working memory. Participants are presented with a number of random and disordered lines, and they must press the number associated with the target end as quickly as possible, identifying the end of a given line. In order to avoid following the lines with their fingers, two keys on the response panel must be pressed during the task.

#### 2.2.4 Signal detection test (SIGNAL)

The SIGNAL test was developed to measure long-term selective attention, which is also related to inhibitory function. The participant must discriminate between relevant and irrelevant stimuli. Among the constantly changing points on the screen, they must detect a square of a certain size from moment to moment. In this way, under time pressure, they inhibit distracting stimuli and respond to relevant stimuli on the response panel.

#### 2.2.5 Executive functions-specific self-assessment

Executive functions-specific self-assessment was assessed using four study-specific single items on a 10-point scale. Single-item self-report measures are commonly used in sport contexts because they provide a quick and practical method for obtaining athletes’ subjective feedback (38). Each item was designed to correspond conceptually to one EF-related domain assessed by the VTS: RT, DT, LVT, SIGNAL. The items were: “How quickly and effectively do you react in a given game situation?” (RT); “How effectively do you make good decisions in different game situations during a match?” (DT); “How effectively do you recognize good passing opportunities?” (LVT); and “How effectively do you concentrate on the pitch while excluding distracting environmental stimuli?” (SIGNAL). The values rated by the players were multiplied by 10, which resulted in a 0–100 scale comparable to VTS scores.

#### 2.2.6 Sports performance

Sports performance is determined by the team's ranking in the given age group championship and the average number of goals scored per championship match (22). In this study, we considered these indicators strictly as descriptive team metrics.

### 2.3 Statistical analysis

Descriptive statistics, including mean and standard deviation, were utilized in this study to explore the characteristics of the sample. Skewness and kurtosis measures were employed to assess the normality of the data. Following the suggestions of Forero (39), we determined that skewness and kurtosis values should fall between −1.5 and 1.5 for normal distribution. A paired sample *t*-test with Cohens’d was conducted to examine the differences between self-assessment and cognitive test scores across all study participants, as well as among different age groups. Additionally, a one-way ANOVA was used to investigate age group differences regarding the study variables. Effect sizes for ANOVA were reported using partial eta squared (ηp^2^). All analyses were performed using Jamovi 3.0 for Mac.

## 3 Results

The mean and standard deviation (SD) were calculated to evaluate the characteristics of the study variables (Table 2). Among the cognitive performance tests, the RT recorded the highest mean score of 69.66 (SD = 24.18), while the SIGNAL had the lowest mean score of 48.38 (SD = 27.26). For self-assessments, the SIGNAL score was the highest at 81.08 (SD = 11.05), whereas the DT self-assessment received the lowest score of 63.84 (SD = 14.87). The skewness and kurtosis values suggested that the distributions were largely symmetrical, albeit with slight negative skewness observed in both the reaction time performance and the stress tolerance self-assessment. However, since all values fell between −1.5 and 1.5, we follow the recommendations of Forero (39) and consider our variables to be normally distributed.

**Table 2:** Mean, standard deviation, skewness and kurtosis of the study variables.

Variable	Mean (SD)	Skewness	Kurtosis
Stress Tolerance Test	54.97 (26.47)	−0.15	−1.08
Focused Attention Test	48.38 (27.26)	0.22	−1.08
Reaction Time Test	69.66 (24.18)	−0.72	−0.40
Visual Perception Test	48.71 (27.07)	0.11	−0.99
Stress Tolerance (Self-Assessment)	63.84 (14.87)	−0.61	0.92
Focused Attention (Self-Assessment)	81.08 (11.05)	−0.47	−0.18
Reaction Time (Self-Assessment)	76.35 (11.77)	0.08	−1.03
Visual Perception (Self-Assessment)	74.32 (14.63)	−0.12	−0.86

Paired samples *t*-tests revealed significant discrepancies between self-assessment and objective cognitive test performance (Table 3). The largest difference was observed in the SIGNAL case, where self-assessment (M = 81.08, SD = 11.05) showed a significantly higher score than the result achieved on the cognitive test (M = 48.91, SD = 27.97), t = 9.41, *p* < 0.001, d = 1.09 (large effect). Similarly, participants significantly overestimated their LVT abilities (self-assessment: M = 74.38, SD = 14.72; test: M = 47.49, SD = 27.44), t = 7.89, *p* < .001, d = 0.90 (large effect). A smaller but significant difference was found for DT (self-assessment: M = 63.84, SD = 14.87; test: M = 54.48, SD = 25.38), t = 2.65, *p* < .01, d = 0.31 (small effect). No significant difference emerged for RT (*p* > .05).

**Table 3:** Results of paired sample *t*-test between self-assessment and cognitive tests.

Variable	Self-assessment (M; SD)	Cognitive test (M; SD)	*t*-test	Cohen's d
Stress Tolerance	63.84 (14.87)	54.48 (25.38)	2.65**	0.31
Focused Attention	81.08 (11.05)	48.91 (27.97)	9.41***	1.09
Reaction Time	76.35 (11.77)	70.95 (22.18)	1.86	0.22
Visual Perception	74.38 (14.72)	47.49 (27.44)	7.89***	0.90

**p < .01, ***p < .001.

Paired comparisons between self-assessments and cognitive test performance across age groups revealed several notable patterns (Table 4). SIGNAL was significant across all age groups, with particularly large effects in younger adolescents (U14: t = 6.98, *p* < .001, d = 1.60; U15: t = 4.02, *p* < .05, d = 1.11; U16: t = 2.69, *p* < .05, d = 0.90; U17: t = 4.87, *p* < .001, d = 1.40; U19: t = 3.31, *p* < .01, d = 0.72). LVT showed similar patterns, with significant effects in U14 (t = 5.28, *p* < .001, d = 1.27), U15 (t = 3.70, *p* < .05, d = 1.03), and U19 (t = 4.67, *p* < .001, d = 1.02) groups. DT demonstrated more variable results, with significant results in U15 (t = 2.60, *p* < .05, d = 0.72) and U19 (t = 4.78, *p* < .001, d = 1.07), but significantly better than self-assessment in U16 (t = −2.01, *p* < .05, d = −0.67). RT showed no significant differences in most age groups. The exception was U17, where test performance exceeded self-assessment (t = −2.20, *p* < .05, d = −0.63). Effect sizes ranged from small (d = 0.28) to very large (d = 1.60), with the largest effects consistently appearing in the SIGNAL domain.

**Table 4:** Paired sample *t*-test results on self-assessments and cognitive tests separating by age groups.

Age group	Variable	Self-assessment (M; SD)	Test performance (M; SD)	*t*-test	Cohen's d
U14	Stress Tolerance	61.58 (13.02)	47.84 (31.21)	1.97	0.45
	Focused Attention	77.37 (9.33)	34.58 (27.00)	6.98***	1.60
	Reaction Time	72.11 (10.84)	63.26 (28.32)	1.24	0.28
	Visual Perception	77.89 (15.84)	45.05 (26.58)	5.28***	1.27
U15	Stress Tolerance	64.62 (8.77)	49.46 (20.52)	2.60*	0.72
	Focused Attention	87.69 (10.13)	49.54 (34.38)	4.02*	1.11
	Reaction Time	78.46 (12.81)	68.31 (20.08)	1.65	0.46
	Visual Perception	74.62 (15.06)	41.08 (32.00)	3.70*	1.03
U16	Stress Tolerance	56.67 (16.58)	76.33 (17.87)	−2.01	−0.67
	Focused Attention	84.44 (12.36)	51.78 (32.42)	2.69*	0.90
	Reaction Time	74.44 (10.14)	69.89 (23.35)	0.47	0.16
	Visual Perception	68.75 (9.91)	40.50 (30.49)	2.57*	0.91
U17	Stress Tolerance	60.00 (15.95)	62.92 (30.25)	−0.27	−0.08
	Focused Attention	80.83 (11.65)	44.58 (21.41)	4.87***	1.40
	Reaction Time	75.00 (11.68)	84.67 (12.83)	−2.20	−0.63
	Visual Perception	60.00 (9.53)	47.08 (32.84)	1.18	0.34
U19	Stress Tolerance	71.00 (16.51)	49.15 (15.03)	4.78***	1.07
	Focused Attention	79.05 (10.91)	62.71 (20.20)	3.31**	0.72
	Reaction Time	80.48 (12.03)	72.14 (18.66)	1.76	0.38
	Visual Perception	81.43 (11.53)	56.57 (20.08)	4.67***	1.02

*p < .05, **p < .01, ***p < .001.

One-way ANOVA results revealed significant age-related differences in cognitive test performance across several domains (see Table 5). The DT scores differed significantly across the age groups (F = 4.43, *p* < 0.01). Because the self-assessment measure consisted of pilot single-item questions, some participants had missing responses on one or more self-assessment items. Therefore, analyses involving self-assessment were conducted using all available valid item-level responses, and no imputation was applied. *Post-hoc* tests indicated that U16 participants (M = 72.45) significantly higher than U15 (M = 45.26), U14 (M = 47.84), and U19 (M = 50.39). Similarly, the SIGNAL Test showed age differences (F = 4.44, *p* < .01), with *post-hoc* comparisons revealing that U19 participants (M = 65.96) showed better results than all younger groups, including U14 (M = 34.58) and U16 (M = 44.45). The RT was also significantly differed (F = 4.17, *p* < .01). *Post-hoc* tests revealed that U17 participants (M = 85.85) had significantly faster reaction times compared to U14 (M = 63.26). Additionally, U15 (M = 66.61) performed significantly worse than U19 (M = 70.63). For self-assessment measures, significant age variation was found only in the LVT Self-Assessment (F = 6.00, *p* < .001). *Post-hoc* tests showed that U19 participants (M = 81.43) reported significantly higher scores than U17 (M = 60.00) and U15 (M = 74.62). Furthermore, U17 (M = 60.00) and U15 (M = 74.62) also showed significant differences. However, no significant age differences were found for self-assessments of DT, SIGNAL, or RT. All *post-hoc* comparisons are available in Supplementary Figure S1.

**Table 5:** Cognitive tests and self-assessments between the age groups.

Variable	Group	N	M (SD)	F	η^2^p
Stress Tolerance Test	U14	19	47.84 (31.21)	4.43**	0.16
	U15	23	45.26 (22.19)		
	U16	22	72.45 (22.99)		
	U17	13	61.46 (29.43)		
	U19	24	50.38 (20.09)		
Focused Attention Test	U14	19	34.58 (27.00)	4.44**	0.16
	U15	23	47.39 (30.02)		
	U16	22	44.45 (26.33)		
	U17	13	44.46 (20.50)		
	U19	24	65.96 (21.00)		
Reaction Time Test	U14	19	63.26 (28.32)	4.17**	0.25
	U15	23	66.61 (26.93)		
	U16	22	67.77 (24.16)		
	U17	13	85.85 (13.00)		
	U19	24	70.63 (20.12)		
Visual Perception Test	U14	19	45.05 (26.58)	0.53	0.02
	U15	23	49.65 (31.30)		
	U16	21	45.10 (28.12)		
	U17	13	46.46 (31.52)		
	U19	24	55.08 (19.73)		
Stress Tolerance Self-Assessment	U14	19	61.58 (13.02)	2.13	0.11
	U15	13	64.62 (8.77)		
	U16	9	56.67 (16.58)		
	U17	12	60.00 (15.95)		
	U19	20	71.00 (16.51)		
Focused Attention Self-Assessment	U14	19	77.37 (9.33)	2.23	0.11
	U17	12	80.83 (11.65)		
	U16	9	84.44 (12.36)		
	U15	13	87.69 (10.13)		
	U19	21	79.05 (10.91)		
Response Time Self-Assessment	U14	19	72.11 (10.84)	1.51	0.11
	U15	13	78.46 (12.81)		
	U16	9	74.44 (10.14)		
	U17	12	75.00 (11.68)		
	U19	21	80.48 (12.03)		
Visual Perception Self-Assessment	U14	19	77.89 (15.84)	6.00***	0.26
	U15	13	74.62 (15.06)		
	U16	9	68.89 (9.28)		
	U17	12	60.00 (9.53)		
	U19	21	81.43 (11.53)		

**p < .01, ***p < .001; reduced subgroup sample sizes for the self-assessment variables reflect missing responses on the pilot self-assessment items. Analyses were based on available valid responses for each item; missing values were not imputed.

## 4 Discussion

This study examined EFs using both objective tests and subjective self-assessments in a sample of Hungarian youth players across different age groups. The team's league final ranking and the average number of goals scored per match served as descriptive metrics for the teams. The findings indicated that age-related differences were more pronounced in the objective measures of EFs than in self-assessments. Older players, particularly those in the U19 group, generally performed better in focused attention and visual perception. Peak scores in reaction time and stress tolerance were observed in the U17 and U16 groups, respectively. In contrast, self-assessments showed little variation across age groups, with players often rating their abilities higher than their objective test results, especially in focused attention, visual perception, and stress tolerance. These results highlight the significance of developmental differences in understanding the cognitive functioning of young players. Additionally, the four questions created to assess self-perceived EFs were specifically designed for this study and are exploratory in nature. Therefore, they should be analysed with caution, and conclusions drawn from them must be interpreted carefully.

The study generally supports previous research on the development of young football players’ EFs (22–26). In the SIGNAL and LVT assessments, the U19 age group achieved the highest performance, while the U14 age group scored the lowest. This indicates that U19 players are better at recognizing passing opportunities and orienting themselves visually on the pitch compared to players in younger age groups. The enhanced functioning of EFs allows U19 players to quickly process visual information and make appropriate decisions, even under pressure (19, 40). Furthermore, U19 players excelled in maintaining their concentration and ignoring external distractions. This ability to focus helps them manage anxiety and improve decision-making skills. It also reduces reaction time and increases tactical harmony with teammates, both of which are crucial for successful football performance and scoring goals (23, 41). The results of the SIGNAL and LVT tests demonstrate that, within this sample, players’ levels of concentration and visual orientation improved with age.

An unexpected finding was that the U16 age group performed best in the DT. This suggests that they have the strongest ability to make effective decisions in complex, high-pressure game situations. This result contradicts existing literature, which indicates that decision-making abilities and cognitive flexibility typically improve with age. While the U16 players excelled in terms of stress tolerance, it's important to note that factors beyond decision-making skills also contribute to a team's success. These include external variables such as weather conditions, the quality of the pitch, and the level of competition posed by opponents. Another possible explanation for this finding is that, at the time of the study, the U16 players had been playing on a full-sized pitch for two years, which may have enhanced their ability to cope with stress. With three more years ahead to shape their football careers, this transitional developmental period likely had a positive impact on their capacity to handle stressful situations (42).

Another unexpected finding was that the U17 group performed best in the RT, suggesting that they react most quickly in a given game situation. While age and biological maturity are significant factors in reaction time, the type of training and the pace of individual development also play an important role in reaction time performance at this age (43, 44). This age group had a reduced number of players because of injuries and withdrawal from competitive sport, thus small-sided training games were prioritized over large-area tactical play (45). These small-sided games specifically focus on developing quick reactions, which helped improve the players’ reaction times. However, it is important to note that the fast reaction times were not accompanied by adequate focused attention. This conclusion is supported by the fact that, among the five age groups in the sample, the U17 group achieved the second-worst score on the SIGNAL test. Although their reaction measurements indicated the fastest responses, they lacked sufficient concentration, which hinders their ability to capitalize on opportunities during real game situations.

The results of the subjective self-assessment indicate that, in this sample, participants consistently rated themselves significantly higher in terms of focused attention, visual perception, and stress tolerance compared to their scores on objective measures of EFs such as the SIGNAL, LVT, and DT tests. This suggests that the players in this sample perceive their abilities to make good decisions during a match (DT), recognize effective passing opportunities (LVT), and concentrate on the field (SIGNAL) as much stronger than what their objective test scores reflect. The only area where participants realistically assessed themselves was in RT, where they accurately recognized how quickly and effectively they could make decisions in specific game situations. Previous research indicates that overestimating one's abilities can negatively impact sports performance and that objective measurements of EFs are generally better predictors of sports performance than self-assessments (18, 26, 33). Following this, the discrepancies between the objective EF tests and the subjective self-assessments were further examined according to age groups.

In the U14 age group, there were two cases (SIGNAL and LVT) where players significantly overestimated their executive functions (EFs) compared to the results obtained from tests. A similar pattern emerged in three cases in the U15 age group (DT, SIGNAL, and LVT), two cases in the U16 age group (SIGNAL and LVT), one case in the U17 age group (SIGNAL), and three cases in the U19 age group (DT, SIGNAL, and LVT). This finding suggests that U17 players are the most realistic in evaluating their stress tolerance, reaction time, and visual perception. The largest discrepancies between self-assessment and EF test results were observed in the U14 and U19 age groups. The differences between subjective self-assessments and objective EF tests may stem from the fact that these methods measure different aspects of cognitive functioning. While objective EF tests assess specific cognitive processes, self-assessment questionnaires evaluate the metacognitive component of EFs (46). Consequently, young athletes tend to overestimate their cognitive abilities, leading to a significant gap between subjective self-assessment and objective measurements (47). In the fields of sports and talent identification, relying solely on self-assessment questionnaires is insufficient for determining players’ actual cognitive profiles (26, 48). Previous research indicates that other factors may also contribute to unduly high self-assessments; these factors can be considered contextual hypotheses, as the current study did not examine them directly. For example, in the U14 and U19 groups, the natural transition to a higher school level may play a role (49–53). Specifically, for the U14 group, transitioning to a full-size pitch requires a higher level of cognitive ability, and overestimating one's skills may serve as a compensatory mechanism (30, 34, 54). For the U19 group, exceptional performance is crucial for the transition to adult football, and high self-assessment can bolster players’ confidence in their abilities (23, 41).

In examining players’ self-assessments of their executive functions (EFs), the LVT test was the only assessment that revealed a significant difference between age groups. Players in the U19 age group rated their ability to recognize suitable passing opportunities and their visual orientation on the pitch higher than players in the other age groups. This finding aligns with their scores on the LVT test, which were the highest among all the age groups included in the study. This indicates that it is reasonable for players in this age group to rate their visual perception higher than their peers, as their actual performance on the relevant LVT test supports this self-assessment (19, 21). The enhanced functioning of EFs enables players to process visual information more quickly, positively impacting their sports performance. However, it is important to note that U19 players exhibited an inflated self-assessment in the area of visual perception compared to their LVT results. This suggests that they may not have a realistic view of their abilities in this regard, based on their own standards. From a practical standpoint, providing feedback on this discrepancy can help encourage a better balance between self-assessment and actual visual skills. This, in turn, may lead to improved sports performance at both the individual and team levels.

Finally, no significant differences were observed across age groups in self-assessed measures between SIGNAL, DT, and RT. Based on the results, the players in the sample gave themselves similar scores in their subjective self-assessment, regardless of age group, for concentration on the pitch (SIGNAL), decision-making during matches (DT), and reaction speed (RT). This is consistent with the finding that self-assessments of tactical abilities change significantly only among players aged 14–18 who play in attacking positions. In contrast, self-assessments of tactical abilities among defenders and midfielders do not show significant changes during the same period (55). Furthermore, in terms of concentration and stress tolerance, self-assessment scores were excessively high compared to the results of the SIGNAL and DT tests. It is also possible that the consistently high self-assessment of the players participating in this study was influenced by the fact that coaches trained players from the U14 age group according to the same professional principles (49). As a result, talented players in a given age group had the opportunity to train with a higher age group and, in some cases, even play matches. This mobility between age groups fostered equal treatment of younger and older players, which contributed to a consistently high level of self-assessment among the participants. However, overly subjective evaluations from players and coaches can have a negative impact on sports performance, as there may be a gap between perceived abilities and actual skill levels. Encouraging open communication about this discrepancy is essential for establishing realistic performance expectations, which should be backed by objective physical measurement results (6, 17).

The study has several limitations that need to be acknowledged. First, we did not examine sport-specific executive functions (EFs) or their self-assessment based on playing position, even though different positional roles may entail varying cognitive and perceptual demands. For instance, attackers may need to make quick finishing moves, defenders must respond effectively during duels, and midfielders need to adapt continuously to shifting spatial and tactical conditions. Second, the self-assessment measure used in this study consisted of four singular indicators that were specifically designed to approximate the VTS domains, rather than utilizing a previously validated, football-specific instrument. Therefore, findings derived from the discrepancies between self-reported and objectively measured EF-related abilities should be interpreted with caution. Third, assessments were conducted during the summer pre-season, a time when training load, fatigue, and seasonal transition effects may have impacted cognitive performance. Additionally, the study employed a cross-sectional design, which restricts our ability to draw developmental or causal conclusions. Other limitations include a lack of data on biological maturation status, individual playing time, and position-specific match demands, all of which could be relevant to the development of executive functions and self-perception in youth football. Finally, the sample consisted of participants from a single Hungarian football academy, which limits the generalizability of the findings to other academies, competitive levels, and cultural contexts. Future research should investigate the functionality of sport-specific EFs through longitudinal studies, particularly focusing on different positions. Furthermore, it is crucial to explore interventions aimed at developing sport-specific EFs, as these are essential determinants of performance. Therefore, training programs specifically designed to enhance these abilities should be developed.

Overall, our research emphasizes the importance of young players’ self-assessments of their executive functions, as these assessments can significantly affect how they evaluate their own performance. From a sports psychology perspective, it is crucial to recognize the potential risks of young players overestimating their abilities. Encouraging realistic self-assessment can promote continuous psychosocial development. Although self-assessment can enhance performance, it may lead to negative consequences in the long run if players do not have a clear understanding of their own capabilities. This duality underscores the need for a balance between actual abilities and self-perception among young players.

## 5 Conclusions

This study highlights several important findings. First, focused attention, visual perception, stress tolerance, and reaction time, which are related to EFs exhibited different developmental patterns across age groups, with older individuals generally performing better than younger ones. Second, regardless of age group, players tended to overestimate their abilities regarding executive functions such as focused attention, visual perception, and stress tolerance. Finally, identifying the discrepancies between subjective self-assessments and objectively measured cognitive abilities can assist practitioners in providing more targeted feedback and supporting the development of a more realistic self-image.

## Data Availability

The raw data supporting the conclusions of this article will be made available by the authors, without undue reservation.
