# Correction: Age-related differences and discrepancies between objective and self-assessed executive functions in youth academy footballers

**DOI:** 10.3389/fspor.2026.1938221

**Published:** 2026-07-21

**Authors:** Levente József Szántai, Tamás Berki, László Tóth

**Affiliations:** 1 Doctoral School of Sport Sciences, Hungarian University of Sport Sciences, Budapest, Hungary; 2 Department of Physical Education Theory and Teaching Methodology, Hungarian University of Sport Sciences, Budapest, Hungary; 3 Department of Psychology and Sports Psychology, Hungarian University of Sports Science, Budapest, Hungary; 4 Teacher Training Institute, Hungarian University of Sports Science, Budapest, Hungary; 5 Institute of Health Promotion and Sport Sciences, Faculty of Education and Psychology, Eötvös Loránd University, Budapest, Hungary

**Keywords:** age groups, executive functions, football, self-assessment, sport performance

Affiliations “Department of Psychology and Sports Psychology, Hungarian University of Sports Science, Budapest, Hungary” and “Institute of Health Promotion and Sport Sciences, Faculty of Education and Psychology, Eötvös Loránd University, Budapest, Hungary” were omitted from the paper. These affiliations have now been added for author László Tóth.

The original version of this article has been updated.

