# Supplementary material for: Age-related differences and discrepancies between objective and self-assessed executive functions in youth academy footballers
Source: Front Sports Act Living. 2026 Jul 9;8:1711349. doi: 10.3389/fspor.2026.1711349 (PMC13434935; doi:10.3389/fspor.2026.1711349)
Supplement: Supplementary file 1 [file Datasheet1.docx]

Supplementary Figure 1. Forest plot: Tukey Post-Hoc Comparisons sorted by Mean Differences
